# Complete Chloroplast Genome Sequence of the Endemic and Endangered Plant *Dendropanax oligodontus*: Genome Structure, Comparative and Phylogenetic Analysis

**DOI:** 10.3390/genes13112028

**Published:** 2022-11-04

**Authors:** Yong Wang, Jing Yu, Yu-Kai Chen, Zhu-Cheng Wang

**Affiliations:** 1 Ministry of Education Key Laboratory for Ecology of Tropical Islands, College of Life Sciences, Hainan Normal University, Haikou 571158, China; 2 Key Laboratory for Quality Regulation of Tropical Horticultural Plants of Hainan Province, College of Horticulture, Hainan University, Haikou 570228, China; 3 College of Life Sciences, Cangzhou Normal University, Cangzhou 061001, China

**Keywords:** *Dendropanax*, chloroplast genomes, nucleotide diversity, phylogenetic analysis

## Abstract

*Dendropanax oligodontus*, which belongs to the family Araliaceae, is an endemic and endangered species of Hainan Island, China. It has potential economic and medicinal value owing to the presence of phenylpropanoids, flavonoids, triterpenoids, etc. The analysis of the structure and characteristics of the *D. oligodontus* chloroplast genome (cpDNA) is crucial for understanding the genetic and phylogenetic evolution of this species. In this study, the cpDNA of *D. oligodontus* was sequenced for the first time using next-generation sequencing methods, assembled, and annotated. We observed a circular quadripartite structure comprising a large single-copy region (86,440 bp), a small single-copy region (18,075 bp), and a pair of inverted repeat regions (25,944 bp). The total length of the cpDNA was 156,403 bp, and the GC% was 37.99%. We found that the *D. oligodontus* chloroplast genome comprised 131 genes, with 86 protein-coding genes, 8 rRNA genes, and 37 tRNAs. Furthermore, we identified 26,514 codons, 13 repetitive sequences, and 43 simple sequence repeat sites in the *D. oligodontus* cpDNA. The most common amino acid encoded was leucine, with a strong A/T preference at the third position of the codon. The prediction of RNA editing sites in the protein-coding genes indicated that RNA editing was observed in 19 genes with a total of 54 editing sites, all of which involved C-to-T transitions. Finally, the cpDNA of 11 species of the family Araliaceae were selected for comparative analysis. The sequences of the untranslated regions and coding regions among 11 species were highly conserved, and minor differences were observed in the length of the inverted repeat regions; therefore, the cpDNAs were relatively stable and consistent among these 11 species. The variable hotspots in the genome included *clpP*, *ycf1*, *rnK-rps16*, *rps16-trnQ*, *atpH-atpI*, *trnE-trnT*, *psbM-trnD*, *ycf3-trnS*, and *rpl32-trnL*, providing valuable molecular markers for species authentication and regions for inferring phylogenetic relationships among them, as well as for evolutionary studies. Evolutionary selection pressure analysis indicated that the *atpF* gene was strongly subjected to positive environmental selection. Phylogenetic analysis indicated that *D. oligodontus* and *Dendropanax dentiger* were the most closely related species within the genus, and *D. oligodontus* was closely related to the genera *Kalopanax* and *Metapanax* in the Araliaceae family. Overall, the cp genomes reported in this study will provide resources for studying the genetic diversity and conservation of the endangered plant *D. oligodontus*, as well as resolving phylogenetic relationships within the family.

## 1. Introduction

The genus *Dendropanax* belongs to the family Araliaceae, comprising shrubs or trees with approximately 93 species worldwide [1]. The species are distributed around tropical America and eastern Asia, with 16 species spread from the south-western to south-eastern provinces of China [1,2]. In China, plants of the Araliaceae family, including species such as *Panax ginseng*, *Panax notoginseng*, and *Panax quinquefolium*, are often used as traditional Chinese herbal medicines because of their anti-inflammatory, anti-tumour, and antioxidant properties [3]. The genus *Dendropanax* is closely related to the genus *Panax* (*Ginseng*) and is a potential source of medicinal flora [4]. Therefore, most plants of the genus *Dendropanax* are also commonly used as traditional herbal medicines in China with a long history of medicinal use recorded in the “*Chinese Materia Medica*”, the “*Dictionary of Traditional Chinese Medicine*”, the “*National Compilation of Chinese Herbs*”, and other Chinese herbal medicinal texts [5,6]. The primary chemical constituents of plants belonging to the genus *Dendropanax* are polyenes, phenylpropanoids, flavonoids, triterpenoids, and other compounds, which possess biological properties such as anti-inflammatory, anti-tumour, insecticidal, and antioxidant effects, similar to those of the *Panax* species [7,8,9,10]. *D. oligodontus* Merr. and Chun [11] grows within dense forests in tropical valleys at an altitude of approximately 800 m [12]. Current studies indicate that *D. oligodontus* is only distributed in the regions of Baoting, Baisha, Lingshui, and Qiongzhong in Hainan Province [12]. Owing to human activities, the natural distribution of *D. oligodontus* is extremely low, and it is included in both the IUCN Red List and China’s Red List of Threatened Species, rendering it critically endangered (CR) [13,14]. Hence, improvements in the basic research and knowledge on *D. oligodontus* will benefit the conservation of this species, as well as aid in its scientific development and utilisation.

Chloroplasts are plant organelles responsible for photosynthesis and the synthesis of starch, fatty acids, pigments, and proteins. In angiosperms, the chloroplast genome (cpDNA) typically comprises a covalently closed circular structure consisting of two inverted repeats (IRs) along with a large single-copy (LSC) and small single-copy (SSC) region [15]. The cpDNA is simpler in structure compared with the nuclear genome, which facilitates easier screening for low-copy genes. Furthermore, it is highly conserved and maternally inherited, is not subject to interference from genetic recombination, and has a relatively independent evolutionary route [16]. Thus, cpDNA has been widely used for the study of plant genetic diversity, phylogeny, and evolution, as well as species identification and taxonomy [17,18,19]. For instance, genes in the coding regions of cpDNA (e.g., *rbcL*, *mat K*, *ndhF*, etc.) evolve at slow rates and are examined in the phylogenetic analyses of larger taxonomic units [20], whereas non-coding regions such as introns and intergenic regions (IGRs) of the cpDNA show faster evolutionary rates and are applied in lower taxonomic levels [16], common sequence fragments of which are *rpl16*, *rpoC1*, *rps16*, *trnL-F*, etc. [21,22,23]. In addition, some cpDNA genes, including the *rbcL*, *matK*, *psbA-trnH*, and *accD* genes, have been selected as alternative fragments for DNA barcoding because of their high conservation rates and are applied in species identification and plant phylogenetic studies [24,25].

Due to the advances in and decreasing cost of next-generation sequencing (NGS) technologies, chloroplast genomes have become ideal tools for studying plant evolution and molecular ecology. Jansen et al. resolved the phylogenetic relationships of the three earliest-differentiated branches of angiosperms based on 81 common genes from 64 cpDNA [26]. Moore et al. used 83 protein-coding and rRNA genes of 86 spermatophyte cpDNA to resolve phylogenetic relationships within eudicots [27]. Studies examining the cpDNA have also been performed for the analysis and study of the breeding and evolutionary adaptations of major crops [28], such as rice [29,30], cotton [31], sorghum [32], maize [33], wheat [34], and soybean [35]. Reduced genetic diversity leading to a reduction in ecological adaptation is one of the main mechanisms of species endangerment, and an increasing number of studies are utilising cpDNA to study the genetic diversity of endangered species and establish conservation measures [36,37,38,39]. Therefore, the application of cpDNA is not only useful in studies involving species identification and molecular breeding, but also provides a molecular basis for the improvement of important cash crops and horticultural varieties, as well as aiding the conservation of rare and endangered plants.

Owing to the remarkable medicinal and health properties as well as the ecological value of plants belonging to the Araliaceae family, this family has garnered considerable interest in recent years [3,4,5,6,7,8,9,10,40,41]. Particularly, species belonging to the genus *Panax*, including *P. ginseng*, *P. notoginseng*, and *P. quinquefolius*, have been extensively studied [3,40,42,43,44]. However, studies on plants of the genus *Dendropanax* have mostly focused on *Dendropanax morbifera* [4,7,8,45], and little is known about *D. oligodontus*. This species is endemic to Hainan Island [11,12] and is characterised as CR [13]. Therefore, basic research on the speciation and evolution of *D. oligodontus* is crucial for the conservation and development of tropical resources.

Analysis of the cpDNA of *D. oligodontus* is vital for the examination of genetic and phylogenetic evolution as well as its conservation and development. Data corresponding to the cpDNA of the Araliaceae family have been published in the NCBI for 34 species in 12 genera. However, of these, only *D. morbifera* (NC_027607.1) [46] and *D. dentiger* (NC_026546.1) [47] of the genus *Dendropanax* have been reported. Therefore, in this study, the cpDNA of *D. oligodontus* was sequenced and assembled using next-generation sequencing technology, and its genes were annotated and submitted to NCBI (MT909827). Next, the genomic structure, codon usage preferences, RNA editing, and SSR of the species were analysed. Furthermore, 10 closely related species were selected to compare data such as sequencing differences, nucleotide diversity (Pi), evolutionary selection pressure, and the expansion and contraction of IR regions. Finally, a phylogenetic tree was constructed using the cpDNA sequences of 24 species to analyse the phylogeny of *D. oligodontus*. The results of this study will provide a valid reference for the conservation, resource development, and utilisation of this endangered species.

## 2. Materials and Methods

### 2.1. Plant Material and DNA Extraction

Leaf samples were collected from Baoting Li Autonomous County, Hainan Province (18°42′ N, 109°42′ E) in July 2020. Fresh leaves were collected and then stored on ice until returning to the laboratory. In addition, the sample specimens were also preserved in the Herbarium of Hainan Normal University (reference number: WY-202007-1). The chloroplast genomic DNA of *D. oligodontus* leaves was extracted using a modified CTAB method [48]. The quality and concentration of the genomic DNA were evaluated using agarose electrophoresis and NanoDrop 2000 (Thermo Fisher Scientific, Inc., Waltham, MA, USA), and the genomic DNA with the necessary quality was then used for library construction.

### 2.2. Sequencing, Assembly, and Gene Annotation of cpDNA

The genome was sequenced on an Illumina Novaseq 6000 platform (Illumina, San Diego, CA, USA) with 150 bp paired-end reads. For enhanced accuracy in subsequent assembly, the clean read data were obtained after quality filtering and trimming. High-quality reads were de novo assembled into a complete cp genome by SPAdes3.11.0 software, using a k-mer set of 21, 45, 65, 85, and 105 [49]. Finally, the complete cp genome was annotated using PGA [50] with the cp genome of *D. dentiger* (NCBI accession number: NC_026546.1) as a reference, coupled with manual checks and adjustment. Additionally, tRNA genes were identified by the tRNAscan-SE 1.21 program [51]. Annotation of rRNA was performed using the RNAmmer 1.2 Server (http://www.cbs.dtu.dk/services; accessed on 17 August 2020), supplemented by homologous sequence comparison to correct for boundary ranges. After sequence annotation, Sequin was used to edit and generate a file for submission to the GenBank database. The edited GenBank annotation files were submitted to Organellar Genome DRAW (OGDRAW) v1.2 for annotation mapping. 

### 2.3. Repeat Sequence and SSR Analysis

Long repeats were analysed using REPuter software (http://bibiserv.techfak.uni-bielefeld.de/reputer; accessed on 6 April 2022) with repeat sequences ≥ 30 bp. Simple sequence repeats (SSR) of *D. oligodontus* were analysed using MISA software [52] with the following settings: single nucleotide > 10, double nucleotide > 5, triple nucleotide > 4, tetranucleotide > 3, pentanucleotide > 3, and hexanucleotide > 3. The minimum distance between two SSRs was 100 bp.

### 2.4. Analyses of Amino Acid Frequencies, Codon Preferences, and RNA Editing Sites

Codon usage bias analysis and calculation of the RSCU values were performed in the program CodonW v1.4.2 (https://sourceforge.net/projects/codonw; accessed on 8 April 2022). RSCU > 1 indicated that the codon was more frequently used and was a preferred codon, whereas RSCU < 1 indicated low usage, and RSCU = 1 indicated that the codon had no usage preference. Potential RNA editing sites were identified using the PREP-Cp online prediction tool (http://prep.unl.edu; accessed on 9 April 2022) with the cut-off value set as 0.8 [53].

### 2.5. Comparative Genomic Analysis and Nucleotide Diversity in the Chloroplast Genome

To investigate the sequence divergence of the chloroplast genome among the analysed Araliaceae family species, the whole chloroplast genome sequences of the 11 species (*D. morbifera*, *D. dentiger*, *Fatsia japonica*, *Kalopanax septemlobus*, *Metapanax delavayi*, *Brassaiopsis hainla*, *Eleutherococcus gracilistylus*, *Schefflera heptaphylla*, *P. ginseng*, and *P. notoginseng*) were analysed using the mVISTA program (http://genome.lbl.gov/vista/mvista/submit.shtml; accessed on 5 April 2022) in Shuffle-LAGAN mode [54]. For the 11 aligned cp genome sequences, the Pi values of the compared chloroplast genomes were calculated using DnaSp v6.12.03 in the sliding window for DNA polymorphism analysis. The window length was set to 600 bp with a step size of 100 bp.

### 2.6. IR Contraction and Expansion Analysis

To observe the expansion and contraction of the IR regions, the IRscope software (https://irscope.shinyapps.io/irapp; accessed on 1 April 2022) [55] was used to map the four adjacent boundary regions (LSC/IRa, IRa/SSC, SSC/IRb, and IRb/LSC) of the cpDNA for the aforementioned 11 species.

### 2.7. Analysis of Evolutionary Selection Pressure

The protein-coding genes of *D. oligodontus* cpDNA were aligned and formatted using ParaAT2.0 with default parameters. The protein-coding gene sequences annotated in the cpDNA were then analysed separately using KaKs_Calculator v.2.0 based on the YN method, and the value of nonsynonymous substitution rate (Ka)/synonymous substitution rate (Ks) was calculated. Ka/Ks > 1 indicated a positive selection effect for the gene, whereas Ka/Ks = 1 indicated a neutral selection and Ka/Ks < 1 indicated a purifying selection effect.

### 2.8. Phylogenetic Analysis

Homologous single-copy genes from 24 species were selected (see Table S4) and compared using MUSCLE v3.8.1551. Conserved sequences were then extracted using Gblocks v0.91b and concatenated for constructing phylogenetic trees. The best amino acid substitution model was selected using prottest v3.4: HIVb + I + G + F, with Chrysanthemum indicum (NC_020320) as the outgroup. Finally, the phylogenetic tree was constructed using RAxML v8.2.12.

## 3. Results

### 3.1. Basic Characteristics of the Chloroplast Genome of D. oligodontus

**Figure 1:**
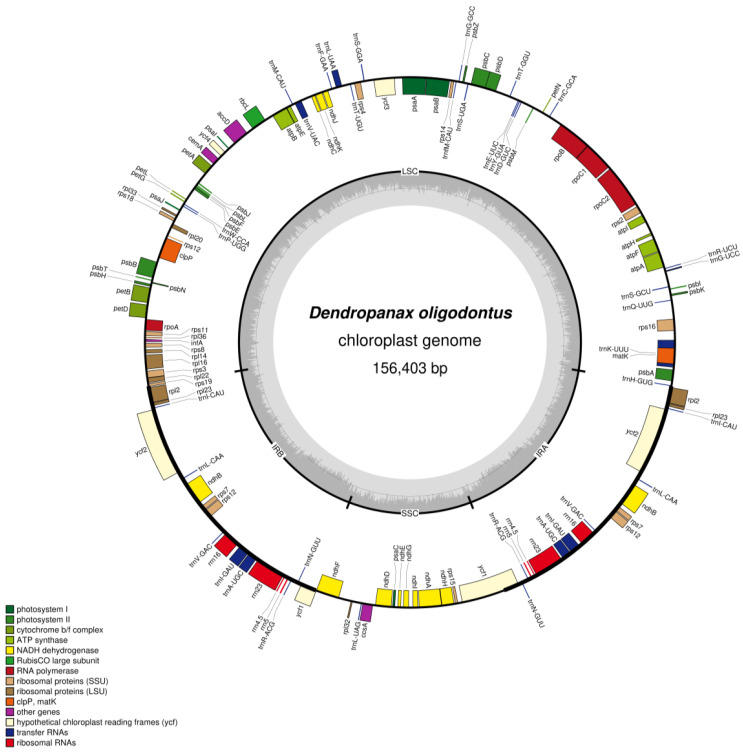
Gene map of the *D. oligodontus* chloroplast genome. Genes shown outside the outer circle are transcribed clockwise, and genes shown inside the circle are transcribed counter-clockwise. Genes belonging to different functional groups are colour-coded. The dashed area in the inner circle indicates the GC content of the chloroplast, and the light grey area corresponds to the AT content of the chloroplast.

After filtering the raw reads, we obtained 4.40 G clean base sequence data (total 29,341,394 reads) with a Q20 of 97.83% using the Illumina Novaseq 6000 platform, and the average sequencing depth was 266.5X. A total of 502,541 reads were mapped to the reference genome, and 25 contigs were assembled. The total length of contigs was 195,086 bp and the largest contig length was 86,440 bp, with N50 value of 25,944 bp and N90 value of 4663 bp. Similar to the structure of angiosperm cpDNA, *D. oligodontus* cpDNA also has a typical quadripartite structure and comprises LSC, SSC, and two IR regions (Figure 1), with a total length of 156,403 bp and a GC content of 37.99%. The LSC region had a length of 86,440 bp (GC%: 36.18%). The SSC region was 18,075 bp long (GC%: 32.07%), and both reverse repeat regions (IRA and IRB) had 25,944 bp (GC%: 43.06%).

The *D. oligodontus* chloroplast genome contains a total of 131 genes (Table 1), including 86 protein-coding genes, 8 rRNA genes, and 37 tRNAs. Of these, seven protein-coding genes (*ndhB*, *rpl2*, *rpl23*, *rps7*, *rps12*, *ycf1*, and *ycf2*), seven tRNA-coding genes *(trnI-CAU*, *trnL-CAA*, *trnV-GAC*, *trnI-GAU*, *trnA-UGC*, *trnR-ACG*, and *trnN-GUU*), and four rRNA-encoding genes (*rrn4.5*, *rrn5*, *rrn16*, and *rrn23*) were in the IR region, and two copies were identified. In addition, *atpF*, *petB*, *petD*, *ndhA*, *ndhB*, *rps16*, *rpl2*, *rpl16*, *rpoC1*, *trnK-UUU*, *trnG-UCC*, *trnL-UAA*, *trnV-UAC*, *trnI-GAU*, and *trnA-UGC* each had one intron, and *rps12*, *clpP,* and *ycf3* had two introns. The *rps12* gene had undergone trans-splicing.

### 3.2. Analysis of Long Repeats and Simple Sequence Repeats

Long-repeat sequence analysis of *D. oligodontus* cpDNA using the REPuter software revealed a total of 13 repetitive sequences, including five forward repeats and eight palindromic repeats (Table 2). The repetitive sequences ranged in length from 30 to 48 bp and were primarily localised on the gene *ycf2*. As many as eight of the sequences were present in two forward repeats (48 bp and 30 bp) localised in the IRA and IRB regions, respectively, and four palindromic repeats (48 bp and 30 bp) present in both IRA and IRB regions. The *ndhA* gene was distributed in one forward repeat (IRB; SSC) and one palindromic repeat (SSC; IRA) region. Both regions were 34 bp in length and located within the intron region. The intergenic spacer (IGS) region of the trnS-GGA gene contained one 30 bp palindromic repeat sequence. In addition, one 44 bp and one 40 bp palindromic repeat sequence were distributed in the LSC and SSC regions, respectively (Table 2).

**Figure 2:**
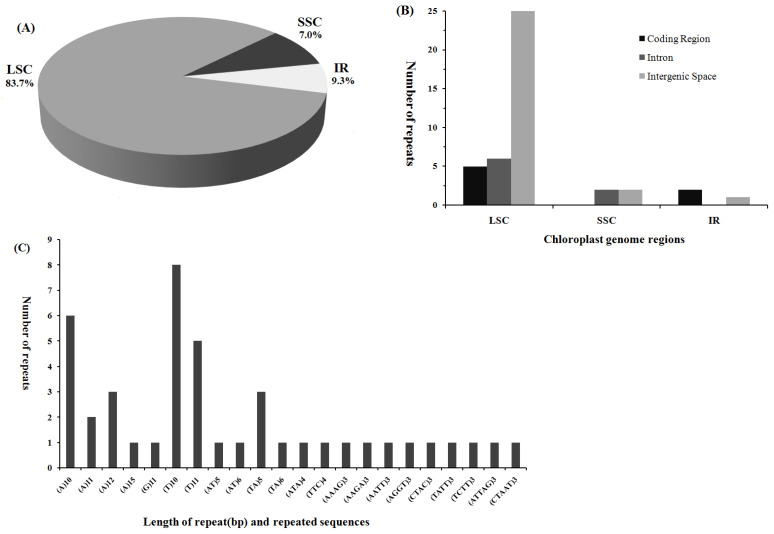
Distribution, type, and presence of simple sequence repeats (SSRs) in the chloroplast genome of *D*. *oligodontus*. (**A**) Presence of SSRs in the large single-copy (LSC), small single-copy (SSC), and inverted repeat (IR) regions. (**B**) Presence of SSRs in the protein-coding regions, introns, and intergenic spacers of LSC, SSC, and IR regions. (**C**) Presence of polymers in the chloroplast genome.

Screening of *D. oligodontus* cpDNA using the MISA software revealed 43 SSR loci, including 26 single-nucleotide repeat types (primarily A or T base repeats, with only one G base repeat), six dinucleotides, two trinucleotides, seven tetranucleotides, and two pentanucleotides (Figure 2C, Supplementary Table S1). The most common loci types were (T)10, (A)10, (T)11, (A)12, and (TA)5. The SSR loci of *D. oligodontus* cpDNA were mainly distributed in 36 loci in the LSC region (83.7%), 3 loci in the SSC region (7.0%), and 4 loci in the IR region (9.3%) (Figure 2A,B). In addition, most SSR loci, as many as 27, were distributed in the IGS region of cpDNA, 6 SSR loci were present in the intron (*atpF*-intron, *rpoC1*-intron, *trnV-UAC*-intron, and *clpP*-intron), and 10 SSR loci were present in the gene coding region, including *rpoC2*, *rpoC1*, *rpoB rpoA*, *ycf1*, *rrn23*, *atpB*, and other genes (Supplementary Table S1).

### 3.3. Codon Usage Bias

*D. oligodontus* cpDNA has 26,514 codons encoding a total of 20 amino acids (without stop codons). Codon classification was performed. The results showed that the most commonly encoded amino acid was leucine (Leu) with 2805 codons (10.58%) and included 6 synonymous codons, with UUA being the most common codon (Table 3, Supplementary Figure S1). This was followed by isoleucine (Ile, 8.39%), serine (Ser, 7.72%), glycine (Gly, 6.92%), arginine (Arg, 6.03%), and phenylalanine (Phe, 5.62%). Cysteine (Cys, 1.11%) was the least-encoded amino acid. Among these codons, the most frequent was lysine (Lys) encoded by AAA (with 1054 codons), and the least frequently used codon was UGC encoding cysteine (with 79 codons) (Table 3). A total of 30 codons had RSCU values higher than 1, indicating that they had codon usage preferences (Table 3). In the third position, 16 codons ended in U(T), 13 ended in A, and only one ended in G, indicating that the codons of *D. oligodontus* cpDNA had a strong A/T preference in the third position. In addition, both the start codon AUG and the Trp-encoding codon UGG had RSCU values equal to 1, indicating no preference, whereas the termination codon UAA had an RSCU value greater than 1, indicating a preference.

### 3.4. Prediction of RNA Editing Sites in Protein-Coding Genes

The Prep-Cp software was used to predict the RNA editing sites for 86 protein-encoding genes of *D. oligodontus* cpDNA (Supplementary Table S2). Of these, RNA editing was observed in 19 genes with a total of 54 editing sites, all of which were C-to-T transitions. In addition, 6 (11.11%), 48 (88.89%), and 0 editing sites were found at the first, second, and third positions of the codon, respectively. These editing sites were primarily present in the genes *ndhB* (nine sites), *ndhD* (eight sites), *ndhA* (five sites), *rpoB* (five sites), *matK* (four sites), and *accD* (three sites), whereas the remaining genes demonstrated one or two sites (Supplementary Table S2). The most common transition induced by site editing was the transition of serine codon TCA (S) to leucine codon TTA (L) (37.04%), followed by proline (P) to leucine (L) (9.2%). H (histidine) to Y (tyrosine) and S (serine) to F (phenylalanine) both showed a frequency of 7.4%. Lower frequencies were observed for R (arginine) to W (tryptophan), P (proline) to L (leucine), T (threonine) to I (isoleucine), P (proline) to S (serine), T (threonine) to M (methionine), A (alanine) to V (valine), L (leucine) to F (phenylalanine), and other transitions.

### 3.5. Comparative Analysis of the Chloroplast Genomes of 11 Araliaceae Species

Using *D. oligodontus* cpDNA as a reference, the cpDNA of 10 plants from eight genera of the Araliaceae family (including two species of the genus *Dendropanax*, two species of the genus *Panax*, and one species each in the genera *Eleutherococcus*, *Schefflera*, *Fatsia*, *Kalopanax*, *Metapanax*, and *Brassaiopsis*) were selected for comparative analysis (Table 4). The cpDNAs of 11 species showed sizes ranging from 155,613 bp (in *Fatsia japonica*) to 156,770 bp (in *Eleutherococcus gracilistylus*), with a maximum difference of 1157 bp and a minimum difference of 10 bp. The size of the LSC region ranged from 86,111 bp (in *P. notoginseng*) to 86,729 bp (in *E. gracilistylus*), with a maximum difference of 618 bp and a minimum difference of 21 bp. The size of the SSC region ranged from 17,867 bp (in *F. japonica*) to 18,554 bp (in *P. notoginseng*), with a maximum difference of 687 bp and a minimum difference of 5 bp. The size of the IR region ranged from 25,629 bp (in *F. japonica*) to 26,074 bp (in *P. ginseng*), with a maximum difference of 445 bp and a minimum difference of 3 bp. Comparative analysis revealed that the GC content of cpDNA in all 11 species was approximately 37.9%, with a maximum difference of only 0.19%. Eight species (*D. morbifera*, *D. dentiger*, *F. japonica*, *Kalopanax septemlobus*, *Metapanax delavayi*, *Brassaiopsis hainla*, *E. gracilistylus* and *P. notoginseng*) had the same total gene number, which was 132 for each, including 87 protein-coding genes, 37 tRNAs and eight rRNA genes (Table 4). The other three species, *D. oligodontus*, *P. ginseng*, and *Schefflera heptaphylla*, had 131, 129, and 123 genes, respectively. They shared eight rRNA genes, too. However, compared with the nine species mentioned above, *D. oligodontus* lacked the *ycf15* gene in the PCGs; *P. ginseng* lacked *petN*, *rpl2* and *trnG-UCC*; and *S. heptaphylla* lacked *psbN*, *trnK-UUU*, *trnG-UCC*, *trnL-UAA*, *trnV-UAC*, two *trnI-GAC,* and two *trnA-UGC*.

**Figure 3:**
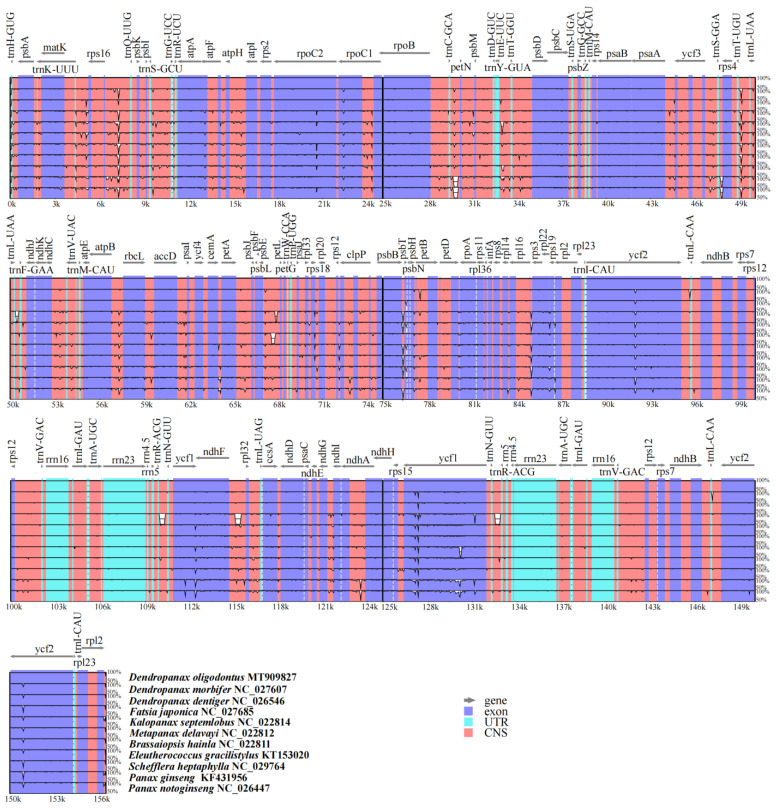
Visualisation alignment of the chloroplast genome sequence of 11 species. The identity percentages are shown on the y-axis and range from 50% to 100%, while the horizontal axis shows the position within the chloroplast genome. Each arrow indicates the annotated genes and the direction of their transcription in the reference genome. Genome regions, i.e., exons, untranslated regions (UTRs), conserved noncoding sequences (CNS), and mRNA, are colour-coded.

Using the mVISTA program for the visual display of multiple-sequence alignment, a comparative analysis showed that the sequences of the untranslated region (UTR) was highly conserved among the 11 Araliaceae species, with some differences in the sequences of the non-coding and exon regions (Figure 3). However, the non-coding sequences showed more differences than the exon sequences; for example, at about 4–11 kb, 28–34 kb, 49–50 kb, etc. (Figure 3). Three species of the genus *Dendropanax* (*D. oligodontus*, *D. morbifera*, and *D. dentiger*) and two species of the genus *Panax* (*P. ginseng* and *P. notoginseng*) had high intra-genus plant sequence similarity, and *F. japonica*, a plant of the genus *Fatsia*, showed more differences in some non-coding regions when compared with other species.

**Figure 4:**
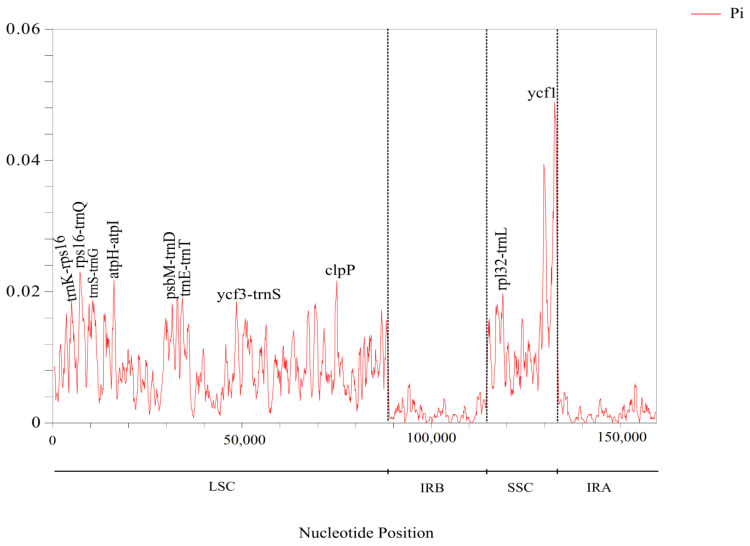
Nucleotide diversity (Pi) analysis of 11 species’ chloroplast genes.

The nucleotide diversity (Pi) of cpDNA in 11 Araliaceae species was analysed using DnaSP software, as shown in Figure 4. The Pi values ranged from 0 to 0.04882, with an overall genomic Pi mean value of 0.00639. Regions with high nucleotide diversity were distributed in the LSC and SSC, and the vast majority were present in the spacer regions of genes, such as *trnK–rps16*, *rps16–trnQ*, *atpH–atpI*, *trnE–trnT*, *psbM–trnD*, *ycf3–trnS*, and *rpl32–trnL*. The genes *clpP* and *ycf1* had relatively higher Pi values (Figure 4). The mean Pi values of the LSC and SSC regions were 0.00854 and 0.01056, respectively. The IR region had lower nucleotide diversity and was more conserved than the single-copy region sequence, with a mean Pi value of only 0.00323.

### 3.6. IR Contraction and Expansion Analysis

**Figure 5:**
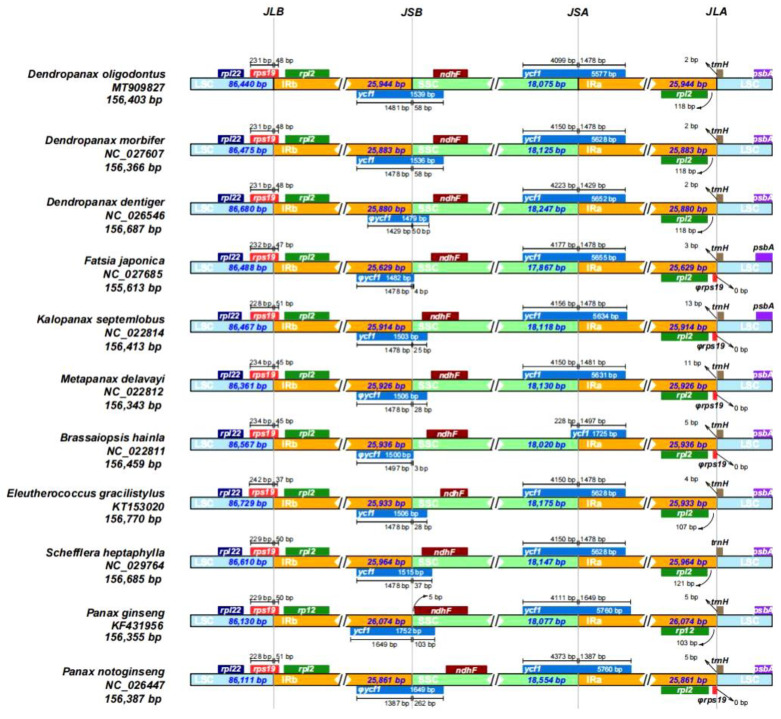
Comparison of the boundaries of the LSC, SSC, and IR regions of the 11 chloroplast genomes. JLB: junction between LSC and IRb; JSB: junction between SSC and IRb; JSA: junction between SSC and IRa; JLA: junction between LSC and IRa.

Variations in the sizes of cpDNA between species are closely related to the expansion and contraction of the IR and SC (LSC and SSC) regions, which is a relatively common phenomenon in cpDNA evolution and may reflect phylogenetic relationships to a certain extent. As shown in Figure 5, the JLB boundaries of all species were located within the *rps19* gene. The base lengths of the *rps19* gene sequences distributed in the LSC (231 bp) and IRb (48 bp) regions were identical in the three species of the genus *Dendropanax*, and differed by only 1 bp in the two species of the genus *Panax* (Figure 5). The results were slightly different for the *rps19* sequence of *E. gracilistylus*, which had only 37 bp distributed in the IRb region, whereas all other species had between 45 to 51 bp. The JSB boundaries of 11 species were all located in the *ycf1* pseudogene (*D. dentiger*, *F. japonica*, *M. delavayi*, *B. hainla*, and *P. notoginseng*) or the *ycf1* gene (*D. oligodontus*, *D. morbifera*, *K. septemlobus*, *E. gracilistylus*, *S. heptaphylla*, and *P. ginseng*), whereas the JSA boundaries of 11 species were all in the *ycf1* gene (Figure 5). In addition, the number of bases at the JSB boundary of the *ycf1* pseudogene or *ycf1* gene deep within the SSC region varied greatly ranging from as low as 3 bp (*B. hainla*) and 4 bp (*F. japonica*) to as high as 262 bp (*P. notoginseng*) (Figure 5). Moreover, the *ndhF* gene of 11 species was distributed in the SSC region. However, it was closely adjacent to the JSB boundary (only 5 bp) in the *P. ginseng* species. Except for the *ycf1* gene of *B. hainla*, which was only 1725 bp long, the *ycf1* genes of 10 species were between 5577 to 5760 bp in length at the JSA boundary. The *ycf1* gene sequences distributed within the IRA region were between 1387 to 1497 bp in length with a smaller difference. At the JLA boundary, the *rpl2* gene (118 bp from the boundary) and the *trnH* gene (2 bp across the boundary) were identical in the three plants of the genus *Dendropanax*, whereas the length of the *trnH* gene across the boundary in the remaining species ranged from 0 to 13 bp. Finally, the *rps19* pseudogene was observed at the JLA boundary in some species (Figure 5).

### 3.7. Analysis of Evolutionary Selection Pressure

During plant evolution, Ka, Ks, and the ratio of Ka/Ks are commonly used to evaluate the rate of evolution between gene sequences and to better elucidate whether selection pressure is associated with a particular protein-coding gene. In this study, the results (Table S3) showed that most of the genes of *Dendropanax* species without Ka/Ks values indicated more conservation, whereas some genes such as *ycf1/2*, *nadA/F*, and *rpoA/B/C1/C2* showed neutral or purifying selection trends. Compared with the species outside the genus *Dendropanax*, *atpF* (except vs. *P. ginseng*), *clpP* (vs. *P. ginseng* and *P. notoginseng*), *matK* (vs. *K. septemlobus*), *ndhA* (vs. *P. ginseng*), *petB* (vs. *S. heptaphylla*), *rpl22* (vs. *E. gracilistylus* and *F. japonica*), *rps12* (vs. *K. septemlobus*, *M. delavayi*, and *B. hainla*) and *ycf1/2* (vs. *F. japonica*, *B. hainla*, *S. heptaphylla*, *P. ginseng*, and *P. notoginseng*) in *D. oligodontus* showed positive selection effects (Table S3). A few genes had values close to 1, indicating a slow evolutionary rate and some conservation in *D. oligodontus* cpDNA.

### 3.8. Phylogenetic Analysis

**Figure 6:**
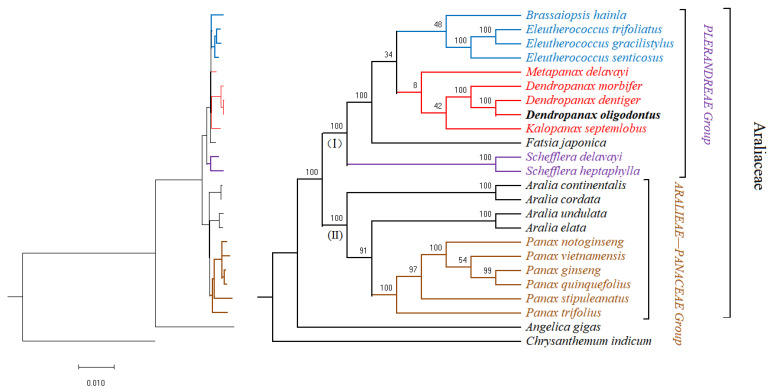
Phylogenetic trees of the Araliaceae species inferred from maximum likelihood (ML) analyses based on the chloroplast genome constructed using coding region data. Support for branches is given by bootstrap values.

To analyse the phylogenetic position of *D. oligodontus* in Araliaceae, a phylogenetic tree was reconstructed for 22 species of the family Araliaceae (see Table S4) using the 54 common protein-coding genes among the 22 complete chloroplast genome sequences. The best amino acid substitution model was selected using prottest v3.4: HIVb + I + G + F, with *Chrysanthemum indicum* of the Asteraceae family and *Angelica gigas* of the Apiaceae family (Apiales) as an outgroup cluster. The results showed that the entire family Araliaceae was clustered into one large branch. Since the Araliaceae family also belongs to Apiales, *A. gigas* was clustered with the Araliaceae family as an extra-family taxon. The Araliaceae taxon was divided into two evolutionary branches (Figure 6, I and II). Branch I included species of the Plerandreae Group, containing 12 species of seven genera. *D. oligodontus* was most closely related to *D. dentiger*, and together with *D. morbifera* were clustered into one branch as the genus *Dendropanax*. Branch II represents the Aralieae–Panaceae group, which includes 10 species of the genera *Panax* and *Aralia*.

## 4. Discussion

### 4.1. Basic Characteristics of the D. oligodontus Chloroplast Genome

Chloroplast DNA typically exists as a double-stranded circular molecule, and the cpDNA of most higher-order plants is a highly conserved tetrameric structure comprising an LSC (approximately 81–90 kb long), SSC (length 18–20 kb), and two IR sequences, IRa and IRb (approximately 20–30 kb long) [15,16]. However, a few plants, such as those belonging to the genus *Erodium* [56], *Medicago truncatula*, *Cicer arietinum*, *Trifolium repens*, and other legumes [57], do not show a tetrameric structure because of the loss of an inverted repeat region. In this study, we sequenced and assembled *D. oligodontus* cpDNA for the first time. The analysis results showed a typical tetrameric structure that was 156,403 bp long with an 86,440 bp LSC, 18,075 bp SSC, 25,944 bp IR, and 37.99% GC content. It comprised 131 genes, including 86 protein-coding genes, 37 tRNA genes, and 8 rRNA genes, which is similar to the reported structural features of the cpDNA of *D. morbifera* [46], *D. dentiger* [47], and the genus *Panax* [3,42] in the Araliaceae family. These results indicate that the cpDNA structure of *D. oligodontus* was relatively conserved.

Repeat sequences and SSRs are widely present in plant cpDNA, and their type, number, and distribution vary among various plants [58]. Chloroplast SSRs have been widely used in studies on the genetic diversity and phylogeny of plant populations, as well as population analysis involving structure, classification, and phylogeographic distribution [16,38]. Analysis of *D. oligodontus* cpDNA revealed a total of 13 repetitive sequences and 43 SSR loci, of which the repetitive sequences were mainly distributed in the coding region of the *ycf2* gene (61.5%), the intron region of the *ndhA* gene, and the IGS region of the trnS-GGA gene. Most of the repeats were attributed to simultaneous distribution in the IR region. The SSR loci were mainly distributed in the LSC region (83.7%) and primarily comprised A or T base repeats. These results are similar to the reported repetitive sequences and SSR distribution characteristics of the chloroplast genomes of nine species in the Araliaceae family, including plants of the genera *Brassaiopsis*, *Eleutherococcus*, *Kalopanax*, and *Macropanax* [59,60]. These species had 35 to 46 cpDNA SSR loci, mostly including single-nucleotide repeats (A/T), and these loci were mainly present on the IGS and intron sequences [59,60]. These data may provide useful genetic references for further research into the molecular ecology of this species.

### 4.2. Gene Codon Usage and RNA Editing Site Prediction in the D. oligodontus Chloroplast Genome

Plant codons exhibit certain usage preferences (codon usage bias, CUB), which are considered an important evolutionary feature of the genome. RSCU is often used as an important indicator of codon preference [61]. Codon usage bias is related to factors such as GC content, tRNA abundance, and protein structure, and mutation and natural selection are the dominant factors affecting the degree of codon bias. The examination of codon preferences is beneficial for understanding phylogenetic patterns in particular species [61,62]. In this study, it was shown that Leu was the most predominant amino acid in *D. oligodontus* cpDNA, and 29 of the 30 codons with an RSCU value higher than 1 ended in A/U, indicating that the codons of *D. oligodontus* cpDNA had a strong A/T preference in the third position. This result is identical to that of the chloroplast genes of *Panax* species belonging to the same family, which also mostly ended in A/T at the third base [59,60]. This is also consistent with the results of previous studies showing that dicotyledon codons use biased A/T endings, whereas monocotyledon codons use biased G/C endings [63,64].

RNA editing may be yet another conserved mechanism for the expansion of genetic information that has been generated in organisms over long periods of evolution, resulting in increased diversity in gene transcription and function [65]. A single gene sequence can be modified by editing to synthesise multiple different proteins involved in the control of different traits or biological processes. Several RNA edits are also observed in the genomes of cellular organelles (including mitochondria and chloroplasts). For instance, the maize chloroplast gene *rpl2* with the start codon ACG must undergo C-to-T editing to form the correct start codon, ATG, before it can initiate transcription and translation [65]. The physiological functions of chloroplast RNA editing primarily involve gene expression regulation, and the effects are manifested in the protein structure. For instance, RNA editing in the non-coding region is important for mRNA splicing, and RNA editing in the coding region of the gene leads to amino acid changes [66,67]. In this study, 54 RNA editing sites were predicted in 86 protein-coding genes of *D. oligodontus* cpDNA. The sites were distributed across 19 genes. All involved C-to-U transitions, with 11.11% and 88.89% of the editing sites observed at the first and second bases of the codons, respectively (see Supplementary Table S2). The predicted number of sites was higher than that observed in an average angiosperm cpDNA, which typically has approximately 30 editing sites [68]. The type of editing observed in this study is consistent with the fact that only C-to-U transitions are found in the chloroplast of higher-order plants, whereas frequent U-to C-transitions are present in the chloroplasts of lower-order plants of the Pteridophyta and Bryophyta taxa [68]. Furthermore, in angiosperms, most editing sites are present in the *ndhB* gene (up to 15). Certain sites are present on several genes, such as *rpoC2*, *rpoB*, *ndhA*, *rpoB*, and *ndhF*, but the number of editing sites is considerably reduced in lower-order plants [66,68]. In the present study, the editing sites in *D. oligodontus* cpDNA were mainly found on the genes *ndhB* (9), *ndhD* (8), *ndhA* (5), *rpoB* (5), *matK* (4), and *accD* (3), which are involved in biological processes such as photosynthetic electron chaining, energy metabolism, gene transcription of proteins, and fatty acid metabolism. These results are consistent with the general characteristics of angiosperms; however, they are also species-specific. In addition, the analysis in this study also revealed that amino acids undergo more than one shift from Ser to Leu after RNA editing, resulting in a shift from hydrophilic to hydrophobic amino acids which increases the hydrophobicity of proteins. These results are in line with the general characteristics of chloroplast RNA in higher-order plants [68].

### 4.3. Comparative Analysis of the Chloroplast Genome of D. oligodontus and Closely Related Species

The size of the cpDNA in angiosperms ranges from 120 to 180 kb, and the size of the IR region ranges from 20 to 30 kb [15,16]. In the present study, statistical analysis of the cpDNA from 11 species belonging to eight genera in the Araliaceae family, including *D. oligodontus* (Table 4), revealed that the genome size ranged from 155.6 to 156.7 kb. The LSC, SSC, and IR regions ranged from 86.1 to 86.7 kb, 17.8 to 18.5 kb, and 25.6 to 26.0 kb, respectively, which was consistent with the characteristics of angiosperm cpDNA [15,16]. In addition, all 11 of these cpDNAs had a GC content of approximately 37.9%, and the total number of genes in most plants was approximately 132 (except *D. oligodontus*, 131; *P. ginseng*, 129; and *S*. *heptaphylla*, 123), which demonstrates the evolutionary conservation among 11 species of the Araliaceae family. Furthermore, the results of multiple-sequence alignment using the mVISTA program has also confirmed the above conclusions. Here, considerable similarities in the genome UTR and coding regions were identified among the cpDNAs of these 11 species, whereas small differential sequences were primarily found in the non-coding regions (Figure 3).

For further identification of highly diverse regions, nucleotide polymorphism (Pi) analysis in the cpDNA of 11 species was conducted using DnaSP software. The results (Figure 4) showed that regions with high Pi values were most distributed in the IGSs of the LSC and SSC. The average value of Pi in the LSC and SSC regions was higher than that in the IR region. We also identified some mutational hotspots, and these highly variable fragments might be adopted as appropriate loci for population genetic and phylogeographic studies.

In addition, a general evolutionary phenomenon in the cpDNA involves expansion and contraction events in the four IR boundaries, which leads to variations—to a considerable extent—in the entire cpDNA of the same or different plant groups. For example, the IR region of the genus Pelargonium is up to 75 kb in length, and the IR regions of *Cryptomeria japonica* and *Pisum sativum* are lost [18,23]. In the present study, small differences were observed in the length of the IR regions in the cpDNA of 11 species from eight genera. These results indicated that the cpDNA of the Araliaceae family did not show such changes in the IR regions, which were relatively stable and consistent (Figure 5). However, minor differences in the sequence lengths of *rps19* and *ycf1* genes across the IR boundary regions were observed in different species, indicating the diversity of IR/SC boundaries across Araliaceae family species. Moreover, the boundary genes, such as the genes *rps19*, *rpl2*, and *trnH* on the IRA-LSC boundary, showed relatively conserved characteristics within the genus *Dendropanax*.

Ka, Ks, and Ka/Ks values can be used to evaluate the rate of evolution in gene sequences. The analysis of these values can provide a reference for our understanding of the adaptive relationship between gene function and structure. A Ka/Ks value of higher than 1 indicates that the gene is subject to positive environmental selection, which helps to determine if the gene is undergoing adaptive evolution [69]. Therefore, in this study, some genes involved in RNA transcription and redox metabolism (e.g., *nadA/F*, and *rpoA/B/C1/C2*) were probably subjected to neutral or purifying selection among the Araliaceae family (Table S3). Correspondingly, the results also showed that some genes (e.g., *atpF*, *clpP*, *matK*, *ndhA*, *petB*, *rpl22*, *rps12*, and *ycf1/2*) involved in energy metabolism, gene information transfer, and photosynthetic electron transfer likely underwent positive selection effects. Interestingly, the *atpF* gene of the genus *Dendropanax* showed strong positive selection compared with other plants of the Araliaceae family (except *P. ginseng*), especially with Ka/Ks values as high as 36.958 compared with those of *E. gracilistylus* (Table S3). These results are consistent with those of Yin et al. [70] who reported that, when compared with deciduous plants, the *atpF* gene of the evergreen sclerophyll *Quercus aquifolioides* showed strong positive selection, and considerable purifying selection was observed within the evergreen sclerophyll taxon [70]. The gene *atpF* is a subunit of H^+^-ATP synthase, which is important for chloroplast electron transport, photorespiration, and resistance to adversity in plants [71]. *D. oligodontus* is also a tropical evergreen plant, and the results of this study imply that the *atpF* gene may be important in the environmental adaptation and species evolution of *Dendropanax*.

### 4.4. Phylogenetic Analysis of the Genus Dendropanax and the Araliaceae Family

Taxonomic studies performed using plant cpDNA sequences have high resolution and can solve many morphological classification problems. Such studies are now widely performed to explore the phylogenetic relationships between species [16,18]. In the present study, the results showed that most nodes of the phylogenetic tree have also obtained a high support rate. There was a clear developmental relationship between each genus or between each species within a genus. The phylogenetic tree showed that *D. oligodontus* was most closely related to *D. dentiger* within the genus *Dendropanax*. This result is consistent with the geographical distribution of the two species. *D. dentiger* is widely distributed in the southern region of China, whereas *D. oligodontus* is distributed only on Hainan Island. Hence, *D. oligodontus* may have evolved from *D. dentiger* due to long-term geographic isolation. Furthermore, *D. morbifera* is primarily distributed in the regions of the Korean Peninsula and Japan [72] and is more distant from the distribution areas of *D. oligodontus* and *D. dentiger*. Thus, evolutionarily, this is also reflected in the results of the mVISTA comparison between the cpDNA of these three species. For instance, regions with lower similarity were located in *prtB* (at approximately 77 kb), *trnL-CAA* (at approximately 96 kb and 147 kb), and *ycf1* (at approximately 127 kb) (Figure 3). In this study, earlier separation of the genera *Schefflera* and *Fatsia* in the Plerandreae (I) clade, as well as the clustering of the genera *Eleutherococcus* and *Brassaiopsis* into one evolutionary branch, are consistent with the results of Dong et al. [60]. In our results, the genera *Metapanax*, *Kalopanax,* and *Dendropanax* were clustered into one evolutionary branch, but low node support was shown among these three genera in the phylogenetic tree (Figure 6). This result seems to be consistent with the reports of Dong et al. [60] and Kim et al. [46] which show that *Kalopanax* and *Metapanax* were more closely related. However, as indicated by Li et al. [73], the genus *Metapanax* is clustered with the genera *Eleutherococcus* and *Brassaiopsis* into one evolutionary branch, forming a sister relationship with the genera *Kalopanax* and *Schefflera*. These inconsistencies require verification in further studies.

## 5. Conclusions

In conclusion, this study used the Illumina Novaseq 6000 platform to obtain the first complete chloroplast sequences of *D. oligodontus*. Similar to the cpDNA of most higher-order plants, the cpDNA of *D. oligodontus* was present as a circular double-stranded molecule with a tetrameric structure, encoding a total of 131 genes. We conducted a structural feature analysis of *D. oligodontus* cpDNA, including repetitive sequence and SSR site identification, the prediction of RNA editing sites in PCGs, the analysis of codon preference, and multiple comparisons of cpDNA sequences among closely related species. These results provide useful information for studying the phylogeny and conservation of *D. oligodontus*, as well as the phylogenetic analysis, identification, classification, and examination of genetic diversity in the Araliaceae family.

## Figures and Tables

**Table 1:** Genes annotated in the *D. oligodontus* chloroplast genomes.

Category	Function	Group of Genes, Gene Names
Photosynthesis	Subunits of ATP synthase	*atpA*, *atpB*, *atpE*, *atpF* *, *atpH*, *atpI*
	Subunits of the Cytochrome b/f complex	*petA*, *petB* *, *petD* *, *petG*, *petL*, *petN*
	Subunits of NADH dehydrogenase	*ndhA* *, *ndhB* *^1^, *ndhC*, *ndhD*, *ndhE*, *ndhF*, *ndhG*, *ndhH*, *ndhI*, *ndhJ*, *ndhK*
	Subunits of Photosystem I	*psaA*, *psaB*, *psaC*, *psaI*, *psaJ*
	Subunits of Photosystem II	*psbA*, *psbB*, *psbC*, *psbD*, *psbE*, *psbF*, *psbH*, *psbI*, *psbJ*, *psbK*, *psbL*, *psbM*, *psbN*, *psbT*, *psbZ*
	Subunit of rubisco	*rbcL*
Transcription and translation	Ribosomal proteins (SSU)	*rps2*, *rps3*, *rps4*, *rps7*^1^, *rps8*, *rps11*, *rps12* **^1#^, *rps14*, *rps15*, *rps16* *, *rps18*, *rps19*
	Ribosomal proteins (LSU)	*rpl2* *^1^, *rpl14*, *rpl16* *, *rpl20*, *rpl22*, *rpl23* ^1^, *rpl32*, *rpl33*, *rpl36*
	Ribosomal RNAs	*rrn4.5*^1^, *rrn5*^1^, *rrn16*^1^, *rrn23*^1^
	RNA polymerase	*rpoA*, *rpoB*, *rpoC1* *, *rpoC2*
	Transfer RNAs	*trnH-GUG*, *trnK-UUU* *, *trnQ-UUG*, *trnS-GCU*, *trnG-UCC* *, *trnR-UCU*, *trnC-GCA*, *trnD-GUC*, *trnY-GUA*, *trnE-UUC*, *trnT-GGU*, *trnS-UGA*, *trnG-GCC*, *trnfM-CAU*, *trnS-GGA*, *trnT-UGU*, *trnL-UAA* *, *trnF-GAA*, *trnV-UAC* *, *trnM-CAU*, *trnW-CCA*, *trnP-UGG*, *trnI-CAU1*, *trnL-CAA* ^1^, *trnV-GAC* ^1^, *trnI-GAU* *^1^, *trnA-UGC* *^1^, *trnR-ACG* ^1^, *trnN-GUU* ^1^, *trnL-UAG*
	Translational initiation factor	*infA*
Other genes	Subunit of acetyl-CoA-carboxylase (fatty acid synthesis)	*accD*
	c-type cytochrome synthesis gene	*ccsA*
	Envelope membrane protein (carbon metabolism)	*cemA*
	Protease	*clpP* **
	Maturase (RNA processing)	*matK*
	Proteins of unknown function	*ycf1*^1^, *ycf2*^1^, *ycf3* **, *ycf4*

Note: * genes containing one intron, ** genes containing two introns, ^#^ trans-spliced genes, ^1^ gene in the IR region with two copies present.

**Table 2:** Long repeats of *D*. *oligodontus* cpDNA.

ID	Size (bp)	Repeat Start 1	Type	Repeat Start 2	Gene	Region	E-Value
1	48	93,175	F	93,193	*ycf2*	IRB	8.68 × 10^−20^
2	48	93,175	P	149,604	*ycf2*	IRB;IRA	8.68 × 10^−20^
3	48	93,193	P	149,622	*ycf2*	IRB;IRA	8.68 × 10^−20^
4	48	149,604	F	149,622	*ycf2*	IRA	8.68 × 10^−20^
5	44	76,557	P	76,557	IGS	LSC	2.22 × 10^−17^
6	40	118,107	P	118,107	IGS	SSC	5.69 × 10^−15^
7	34	100,238	F	122,852	*ndhA*(intron); IGS	IRB;SSC	2.33 × 10^−11^
8	34	122,852	P	142,573	*ndhA*(intron); IGS	SSC;IRA	2.33 × 10^−11^
9	30	9335	P	47,553	*trnS-GGA*; IGS	LSC	5.97 × 10^−9^
10	30	93,175	F	93,211	*ycf2*	IRB	5.97 × 10^−9^
11	30	93,175	P	149,604	*ycf2*	IRB;IRA	5.97 × 10^−9^
12	30	93,211	P	149,640	*ycf2*	IRB;IRA	5.97 × 10^−9^
13	30	149,604	F	149,640	*ycf2*	IRA	5.97 × 10^−9^

Note: F–forward; P–palindromic; IGS–intergenic space.

**Table 3:** Relative synonymous codon usage (RSCU) for protein-coding genes in *D. oligodontus*.

Codon	AA	ObsFreq	RSCU	Codon	AA	ObsFreq	RSCU	Codon	AA	ObsFreq	RSCU
UAA	*	42	1.47	GAA	Glu	1040	1.5	AUG	Met	619	1
UGA	*	21	0.73	CAA	Gln	695	1.49	UUU	Phe	954	1.28
UAG	*	23	0.8	CAG	Gln	236	0.51	UUC	Phe	535	0.72
GCC	Ala	234	0.66	GAG	Glu	344	0.5	AAG	Lys	374	0.52
GCA	Ala	402	1.14	GGU	Gly	600	1.31	AAA	Lys	1054	1.48
GCU	Ala	620	1.76	GGA	Gly	719	1.57	CCU	Pro	430	1.53
GCG	Ala	153	0.43	GGC	Gly	190	0.41	CCA	Pro	317	1.13
CGC	Arg	88	0.33	GGG	Gly	327	0.71	CCC	Pro	211	0.75
CGA	Arg	383	1.44	CAC	His	140	0.45	CCG	Pro	164	0.58
CGU	Arg	345	1.29	CAU	His	481	1.55	UCU	Ser	580	1.7
AGG	Arg	174	0.65	AUC	Ile	472	0.64	UCA	Ser	409	1.2
AGA	Arg	486	1.82	AUU	Ile	1042	1.4	UCC	Ser	330	0.97
CGG	Arg	124	0.47	AUA	Ile	711	0.96	AGU	Ser	410	1.2
GAU	Asp	871	1.59	CUU	Leu	598	1.28	UCG	Ser	195	0.57
GAC	Asp	222	0.41	UUA	Leu	850	1.82	AGC	Ser	123	0.36
AAU	Asn	967	1.53	CUA	Leu	394	0.84	ACG	Thr	153	0.45
AAC	Asn	295	0.47	CUC	Leu	193	0.41	ACC	Thr	257	0.75
GUC	Val	181	0.51	CUG	Leu	184	0.39	ACU	Thr	544	1.58
GUA	Val	525	1.47	UUG	Leu	586	1.25	ACA	Thr	420	1.22
GUU	Val	513	1.43	UGC	Cys	79	0.54	UAC	Tyr	207	0.42
GUG	Val	211	0.59	UGU	Cys	216	1.46	UAU	Tyr	790	1.58
UGG	Trp	461	1								

Note: * stop codon.

**Table 4:** Summary of the complete chloroplast genome characteristics of 11 Araliaceae species.

Species	GenBank Accession No.	Genome Size (bp)	LSC Size (bp)	SSC Size (bp)	IR Size (bp)	GC Content (%)	Number of Genes	Protein Coding Genes	tRNA Genes	rRNA Genes
*D*. *oligodontus*	MT909827	156,403	86,440	18,075	25,944	37.99	131	86	37	8
*D*. *morbifera*	NC_027607	156,366	86,475	18,125	25,883	37.99	132	87	37	8
*D*. *dentiger*	NC_026546	156,687	86,680	18,247	25,880	37.96	132	87	37	8
*F*. *japonica*	NC_027685	155,613	86,488	17,867	25,629	37.91	132	87	37	8
*K*. *septemlobus*	NC_022814	156,413	86,467	18,118	25,914	37.95	132	87	37	8
*M*. *delavayi*	NC_022812	156,343	86,361	18,130	25,926	37.93	132	87	37	8
*B*. *hainla*	NC_022811	156,459	86,567	18,020	25,936	37.92	132	87	37	8
*E*. *gracilistylus*	KT153020	156,770	86,729	18,175	25,933	37.95	132	87	37	8
*S*. *heptaphylla*	NC_029764	156,685	86,610	18,147	25,964	37.93	123	86	29	8
*P*. *ginseng*	KF431956	156,355	86,130	18,077	26,074	38.08	129	85	36	8
*P*. *notoginseng*	NC_026447	156,387	86,111	18,554	25,861	38.08	132	87	37	8

## Data Availability

The data presented in this study are available on request from the corresponding author. The data are not publicly available due to privacy. The complete chloroplast genome sequence of *D. oligodontus* was deposited at NCBI (GenBank accession number: MT909827).

## Supplementary Materials

The following supporting information can be downloaded at: https://www.mdpi.com/article/10.3390/genes13112028/s1, Figure S1: Codon content of 20 amino acids and stop codons in the *D. oligodontus* cpDNA; Table S1: Summary of the SSRs in chloroplast gene of *D. oligodontus*; Table S2: Prediction of the RNA editing sites in chloroplast genome of *D. oligodontus*; Table S3: The rate of Ka/Ks in the chloroplast genomes of 11 Araliaceae species; Table S4: Plant materials for data matrix.
