# Supplementary material for: Complete Chloroplast Genome Sequence of the Endemic and Endangered Plant Dendropanax oligodontus: Genome Structure, Comparative and Phylogenetic Analysis
Source: Genes (Basel). 2022 Nov 4;13(11):2028. doi: 10.3390/genes13112028 (PMC9690231; doi:10.3390/genes13112028)
Supplement: Supplementary file 1 [file genes-13-02028-s001.zip › genes-1998104-supplementary.pdf]

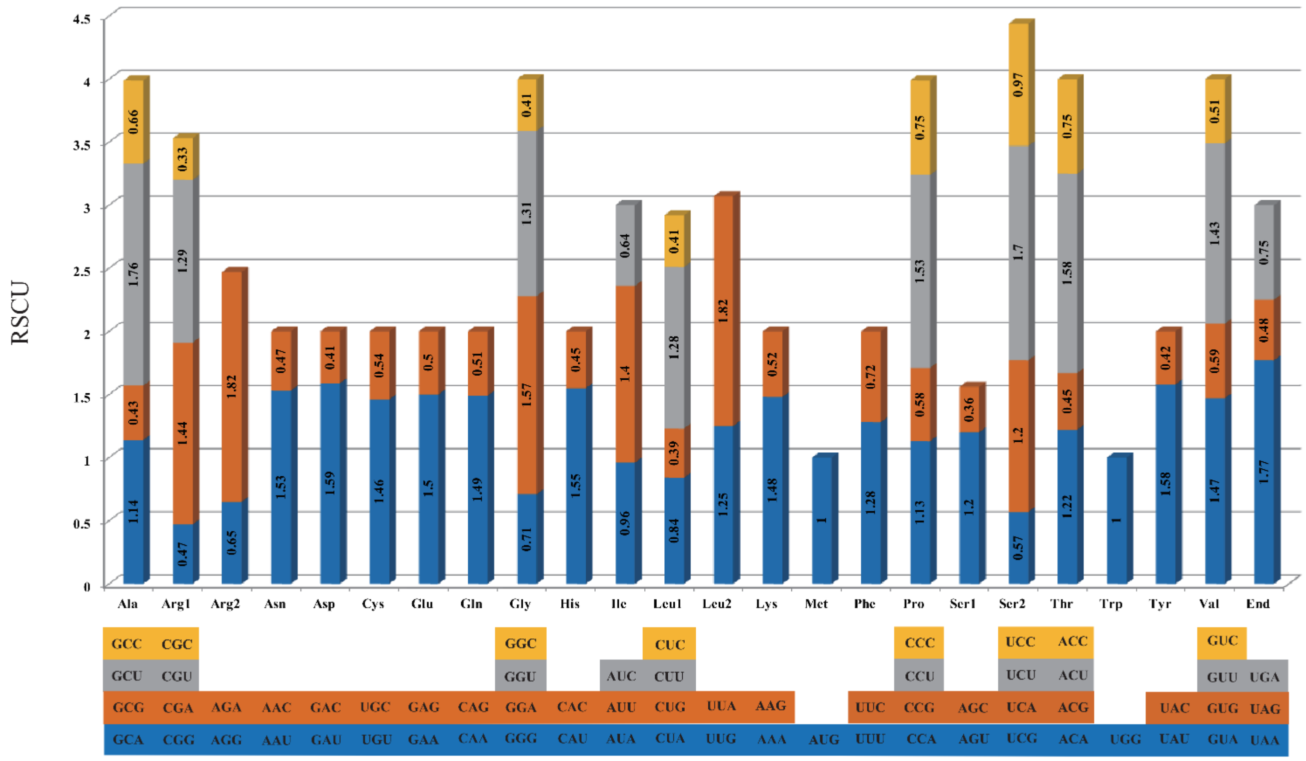

Figure S1. Relative synonymous codon usage (RSCU).

Table S1. Summary of the SSRs in chloroplast gene of *D. oligodontus*

ID	Repeat Motif	Length (bp)	Start	End	Region	*Gene*
1	(A)11	11	443	453	LSC	IGS
2	(A)10	10	4,803	4,812	LSC	IGS
3	(G)11	11	5,105	5,115	LSC	IGS
4	(A)10	10	6,530	6,539	LSC	IGS
5	(A)10	10	9,590	9,599	LSC	IGS
6	(T)10	10	13,522	13,531	LSC	*atpF*-intron
7	(A)12	12	14,144	14,155	LSC	IGS
8	(A)12	12	17,569	17,580	LSC	IGS
9	(T)11	11	19,783	19,793	LSC	*rpoC2*
10	(AT)6	12	22,176	22,187	LSC	*rpoC1*
11	(A)12	12	23,820	23,831	LSC	*rpoC1*-intron
12	(T)10	10	27,485	27,494	LSC	*rpoB*
13	(TA)5	10	29,795	29,804	LSC	IGS
14	(AAGA)3	12	30,918	30,929	LSC	IGS
15	(TCTT)3	12	30,940	30,951	LSC	IGS
16	(AATT)3	12	31,084	31,095	LSC	IGS
17	(TA)5	10	33,493	33,502	LSC	IGS
18	(A)10	10	54,071	54,080	LSC	*trnV-UAC*-intron
19	(T)10	10	56,729	56,738	LSC	*atpB*
20	(ATA)4	12	56,783	56,794	LSC	*atpB*
21	(AT)5	10	59,105	59,114	LSC	IGS
22	(A)10	10	61,426	61,435	LSC	IGS

23	(T)11	11	62,026	62,036	LSC	IGS
24	(TTC)4	12	69,797	69,808	LSC	IGS
25	(TA)6	12	70,117	70,128	LSC	IGS
26	(TATT)3	12	70,158	70,169	LSC	IGS
27	(A)15	15	70,644	70,658	LSC	IGS
28	(T)10	10	71,822	71,831	LSC	IGS
29	(AAAG)3	12	72,512	72,523	LSC	*clpP*-intron
30	(T)11	11	72,812	72,822	LSC	*clpP*-intron
31	(A)11	11	72,975	72,985	LSC	*clpP*-intron
32	(T)10	10	80,412	80,421	LSC	*rpoA*
33	(T)10	10	82,346	82,355	LSC	IGS
34	(T)10	10	83,367	83,376	LSC	IGS
35	(T)11	11	83,455	83,465	LSC	IGS
36	(TA)5	10	86,181	86,190	LSC	IGS
37	(ATTAG)3	15	101,128	101,142	IRB	IGS
38	(AGGT)3	12	107,871	107,882	IRB	*rrn23*
39	(A)10	10	115,111	115,120	SSC	IGS
40	(T)11	11	128,728	128,738	SSC	*ycf1*
41	(T)10	10	130,042	130,051	SSC	*ycf1*
42	(CTAC)3	12	134,960	134,971	IRA	*rrn23*
43	(CTAAT)3	15	141,702	141,716	IRA	IGS

Table S2. Prediction of the RNA editing sites in chloroplast genome of *D. oligodontus*

Gene Name	Strand	Region	Nt Pos	AA Pos	Effect	Score
	+	LSC	125	42	aCg (T) => aTg (M)	1
accD	+	LSC	1243	415	Cca (P) => Tca (S)	1
	+	LSC	1451	484	cCt (P) => cTt (L)	1
atpA	-	LSC	773	258	tCa (S) => tTa (L)	1
	-	LSC	791	264	cCc (P) => cTc (L)	1
atpI	-	LSC	629	210	tCa (S) => tTa (L)	1
	-	LSC	643	215	Cat (H) => Tat (Y)	1
matK	-	LSC	988	330	Ctt (L) => Ttt (F)	0.86
	-	LSC	1010	337	gCg (A) => gTg(V)	0.86
	-	LSC	1246	416	Cat (H) => Tat (Y)	1
	-	SSC	107	36	cCt (P) => cTt (L)	1
	-	SSC	320	107	aCa (T) => aTa (I)	1
ndhA	-	SSC	566	189	tCa (S) => tTa (L)	1
	-	SSC	914	305	aCt (T) => aTt (I)	0.8
	-	SSC	1073	358	tCc (S) => tTc (F)	1
	+/-	IR	95	32	tCa (S) => tTa (L)	1
	+/-	IR	413	138	cCa (P) => cTa (L)	1
	+/-	IR	532	178	Cat (H) => Tat (Y)	1
ndhB	+/-	IR	557	186	tCa (S) => tTa (L)	0.8
	+/-	IR	683	228	cCa (P) => cTa (L)	1
	+/-	IR	692	231	tCt (S) => tTt (F)	1

	+/-	IR	776	259	tCa (S) => tTa (L)	1
	+/-	IR	782	261	tCa (S) => tTa (L)	1
	+/-	IR	1427	476	cCa (P) => cTa (L)	1
ndhD	-	SSC	29	10	aCg (T) => aTg (M)	1
	-	SSC	340	114	Cgg (R) => Tgg (W)	0.8
	-	SSC	410	137	tCa (S) => tTa (L)	1
	-	SSC	626	209	tCa (S) => tTa (L)	1
	-	SSC	701	234	tCg (S) => tTg (L)	1
	-	SSC	905	302	tCa (S) => tTa (L)	1
	-	SSC	914	305	cCc (P) => cTc (L)	1
	-	SSC	1325	442	tCa (S) => tTa (L)	0.8
ndhF	-	SSC	290	97	tCa (S) => tTa (L)	1
ndhG	-	SSC	166	56	Cat (H) => Tat (Y)	0.8
	-	SSC	314	105	aCa (T) => aTa (I)	0.8
petB	+	LSC	418	140	Cgg (R) => Tgg (W)	1
	+	LSC	611	204	cCa (P) => cTa (L)	1
psaI	+	LSC	80	27	tCt (S) => tTt (F)	0.86
	+	LSC	104	35	aCt (T) => aTt (I)	1
psbE	-	LSC	214	72	Cct (P) => Tct (S)	1
psbF	-	LSC	77	26	tCt (S) => tTt (F)	1
rpl20	-	LSC	308	103	tCa (S) => tTa (L)	0.86
rpoA	-	LSC	368	123	tCa (S) => tTa (L)	1
	-	LSC	833	278	tCa (S) => tTa (L)	1
rpoB	-	LSC	338	113	tCt (S) => tTt (F)	1
	-	LSC	473	158	tCa (S) => tTa (L)	0.86
	-	LSC	551	184	tCa (S) => tTa (L)	1
	-	LSC	566	189	tCg (S) => tTg (L)	1
	-	LSC	2426	809	tCa (S) => tTa (L)	0.86
rpoC1	-	LSC	62	21	tCa (S) => tTa (L)	1
rpoC2	-	LSC	2290	764	Cgg (R) => Tgg (W)	1
	-	LSC	3259	1087	Ccg (P) => Tcg (S)	1
rps14	-	LSC	80	27	tCa (S) => tTa (L)	1
	-	LSC	149	50	cCa (P) => cTa (L)	1

Table S3. The rate of *Ka* / *Ks* **in the chloroplast genomes of 11 Araliaceae species**

Genes	*Ka* / *Ks* **Value**									
	D. oligodontus vs D. morbifer	*D. oligodontus vs D. dentiger*	*D. oligodontus vs F. japonica*	*D. oligodontus vs K. septemlobus*	*D. oligodontus vs M. delavayi*	*D. oligodontus vs B. hainla*	*D. oligodontus vs E. gracilistylus*	*D. oligodontus vs S. heptaphylla*	*D. oligodontus vs P. ginseng*	*D. oligodontus vs P. notoginseng*
accD	0.111		0.131	0.117	0.080	0.120	0.228	0.347	0.313	0.213
atpA			0.149	0.092	0.074	0.185	0.092	0.183	0.131	0.136
atpB								0.102	0.210	0.416
atpE				0.683	0.205	0.943				
atpF			1.375	1.651	1.379	1.476	36.958	3.020		3.567
atpI	0.318							0.159		
ccsA	0.267		0.628	0.629	0.808	0.234	0.540	0.358	0.336	0.464
cemA	0.283					0.142	0.280			0.138
clpP			0.301	0.301	0.301	0.301	0.302	0.790	1.323	1.325
matK	0.427		0.515	1.838	0.915	0.547	0.627	1.033	0.779	0.515
ndhA	0.284	0.143	0.142	0.070	0.285	0.743	0.143	0.149	1.144	0.712
ndhB								0.326		
ndhD			0.623	0.207		0.207	0.158	0.125	0.105	0.143
ndhE									0.213	0.071
ndhF	0.233	0.473	0.373	0.363	0.398	0.496	0.302	0.359	0.176	0.209
ndhG				0.270			0.264	0.134	0.178	
ndhH	0.258		0.065	0.056		0.129		0.179	0.059	0.049
ndhI			0.126				0.063		0.040	0.040
ndhJ						0.255	0.259		0.521	0.258
ndhK							0.274		0.039	0.039
petA			0.092	0.139	0.092		0.139	0.047	0.302	0.363
petB								4.083		
petD						0.300				
psaA			0.101	0.086	0.223	0.052	0.057	0.104	0.106	0.108

psaB			0.045	0.064	0.053	0.045	0.054	0.040	0.023	0.030
psbA									0.121	0.086
psbB						0.117	0.054	0.032	0.029	0.026
psbC						0.120	0.111	0.121	0.029	0.081
psbD					0.291		0.145	0.145		
psbH				0.238						
psbJ										0.206
psbK							0.315			
psbL								0.159		
psbZ										0.333
rbcL		0.293	0.563	0.219	0.193	0.288	0.192	0.627	0.444	0.523
rpl16									0.074	0.074
rpl20				0.250					0.158	0.243
rpl22			1.709	0.532			1.361	0.897	0.173	0.149
rpl33			0.292	0.889	0.292	0.292		0.292	0.292	0.292
rpoA	0.649		0.510	0.604	0.950	0.445	0.811	0.539	0.532	0.553
rpoB	0.263		0.124	0.122	0.107	0.193	0.161	0.218	0.042	0.042
rpoC1	0.265		0.174	0.269	0.269	0.387	0.190	0.135	0.038	0.096
rpoC2	0.275		0.229	0.205	0.496	0.980	0.234	0.562	0.256	0.256
rps11									0.336	
rps12				2.840	2.204	2.840		1.089		
rps14							0.320			
rps18								0.254		
rps19							0.059	0.120	0.115	0.115
rps2	0.132		0.528	0.209	0.139	0.528	0.262	0.155	0.124	0.094
rps3									0.258	0.376
rps4								0.282		
rps7						0.249				
rps8			0.143				0.145		0.192	0.289
ycf1	1.032	0.531	2.347	0.778	0.760	1.107	0.715	1.291	1.123	1.098

ycf2	0.289	0.289	1.885	0.629	1.048	1.179	1.068	0.731	1.533	2.157
ycf4			0.146	0.295	0.297	0.100		0.169	0.149	0.199

Table S4. Plant materials for data matrix

Species	GenBank accession
Eleutherococcus senticosus	NC_016430
Eleutherococcus gracilistylus	KT153020
Eleutherococcus trifoliatus	MN727298
Brassaiopsis hainla	NC_022811
Kalopanax septemlobus	NC_022814
Dendropanax morbifer	NC_027607
Dendropanax oligodontus	MT909827
Dendropanax dentiger	NC_026546
Metapanax delavayi	NC_022812
Fatsia japonica	NC_027685
Schefflera delavayi	NC_022813
Schefflera heptaphylla	NC_029764
Aralia undulata	NC_022810
Aralia elata	KT153023
Aralia continentalis	NC_041648
Aralia cordata	NC_040964
Panax notoginseng	NC_026447
Panax vietnamensis	NC_028704
Panax ginseng	KF431956
Panax quinquefolius	NC_027456
Panax stipuleanatus	NC_030598
Panax trifolius	NC_037994
Angelica gigas	KX118044
Chrysanthemum indicum	NC_020320
